# Taxonomic notes on the genus *Rhaphuma* Pascoe, 1858 (Coleoptera, Cerambycidae) in China, with focus on the species complex of *Rhaphuma incarinata* Pic, 1925

**DOI:** 10.3897/zookeys.1283.170982

**Published:** 2026-06-24

**Authors:** Lu Chen, Ye Qi, Xiaoyi Wang, Congrong Jia, Zhu Li

**Affiliations:** 1 College of Plant Protection, Southwest University, Chongqing, China College of Plant Protection, Southwest University Chongqing China; 2 Bishan District Landscaping and Greening Management Office, Chongqing, China Bishan District Landscaping and Greening Management Office Chongqing China

**Keywords:** Cerambycinae, Clytini, new records, new synonymy, *
Rhaphuma
*

## Abstract

Through integrated morphological examination and cytochrome *c* oxidase subunit I (COI) sequences, seven species of the *Rhaphuma incarinata* species complex were recorded from China: *R. incarinata* Pic, 1925, *R. mekonga* Gressitt & Rondon, 1970, *R. phiale* Gahan, 1906, *R. pseudominuta* Gressitt & Rondon, 1970 (= *Rhaphuma aequalis* Holzschuh, 1991, **syn. nov**.), *R. puncticollis* Holzschuh, 1992, *R. quadrimaculata* Pic, 1923 and *R. subvarimaculata* Gressitt & Rondon, 1970. Descriptions of external morphology and male genitalia are provided, and redescriptions of *R. incarinata* and *R. pseudominuta* are given. *Rhaphuma incarinata*, *R. mekonga*, and *R. pseudominuta* are recorded from China for the first time.

## Introduction

The genus *Rhaphuma* Pascoe, 1858, with *Clytus quadricolor* Castelnau & Gory, 1841 as the type species, represents a diverse group within Cerambycidae. It currently comprises 232 species and subspecies distributed in the Palaearctic and Oriental regions, of which approximately 89 taxa are recorded from China (Tavakilian and Chevillotte 2026). Owing to its high species richness and morphological diversity, this genus has long attracted the attention of taxonomists, resulting in a steady increase in the number of described taxa. Notably, Pic (1900, 1915, 1925, 1927, 1937, 1943, 1950 etc.) described 29 taxa between 1900 and 1950, Gressitt and Rondon (1970) recorded 22 species from Laos, including 15 new taxa; and Holzschuh (1983, 1984, 1991, 1992, 2003, 2016, 2018a, 2018b etc.) described 67 taxa over 40 years. More recently, Viktora (2015, 2019, 2020, 2021 etc.) described an additional 46 species over the past decade.

Despite extensive research, the taxonomy of the genus *Rhaphuma* remains problematic. Recent molecular phylogenetic analyses suggest that the genus is polyphyletic (Zamoroka 2021). In addition, many species exhibit highly similar external morphologies, particularly in elytral patterns, which complicates species identification. Gressitt and Rondon (1970) proposed the “*diana*-group”, characterized by short basal elytral stripes and subtransverse blackish bands on the elytra. This informal grouping encompasses a number of species that are superficially similar in appearance; however, it has not been widely adopted in subsequent studies, and its taxonomic validity remains uncertain.

Within this group, several species closely related to *R. incarinata* Pic, 1925 form a subset characterized by highly similar external morphologies and overlapping diagnostic features, rendering them difficult to distinguish using traditional characters alone. This subset, here treated as the *R. incarinata* species complex, includes *R. phiale* Gahan, 1906, *R. quadrimaculata* Pic, 1923, *R. incarinata* Pic, 1925, *R. subvarimaculata* Gressitt & Rondon, 1970, *R. improvisa* Holzschuh, 1991 and *R. puncticollis* Holzschuh, 1992.

Moreover, important diagnostic characters, particularly those of the male genitalia, remain insufficiently documented for several taxa within this species complex, likely leading to frequent misidentifications. Molecular data, which have proven useful in resolving species boundaries in Cerambycidae (Grebennikov et al. 2017; Pérez-Flores and Toledo-Hernández 2022), remain scarce for this genus. As a result, species boundaries within the *R. incarinata* group remain poorly defined due to insufficient comparative study of genital morphology and a lack of molecular data.

The present study aims to clarify the taxonomic relationships among morphologically similar species related to *Rhaphuma incarinata* Pic, 1925. We re-examine diagnostic characters, with particular emphasis on male genitalia and mitochondrial COI sequences, in order to delimit species boundaries and provide reliable criteria for distinguishing closely related taxa. This study provides a framework for species delimitation within *Rhaphuma* and contributes to a better understanding of its taxonomic structure.

## Material and methods

### Morphological examination

All materials examined in the present study are deposited in the Insect Collection of Southwest University, Chongqing, China (SWU).

Specimens were preserved in 100% ethanol. Male genitalia were prepared by softening the specimens in hot water for several minutes. The genitalia were carefully removed from the abdomen and cleared in 10% NaOH for several minutes. After examination, the genital structures were preserved in glycerin in microvials and pinned beneath the corresponding specimens.

Photographs of adult habitus were taken using a Canon 7D digital camera in combination with Helicon Remote v. 2.2.7 and Helicon Focus v. 5.2 (Helicon Soft Limited, Kharkov, Ukraine). Male genitalia were photographed using a Leica M205A stereomicroscope.

### Distribution maps

Distribution maps were generated using two standard base maps: a China base map (GS (2024) 0650) and a world base map (GS (2016) 1666). Both unmodified base maps were obtained from the Standard Map Service website of the National Bureau of Surveying and Mapping Geographic Information (http://bzdt.ch.mnr.gov.cn/index.html). All geographic data processing and map visualization were performed using ArcGIS v. 10.8.1.

### Taxon sampling, DNA extraction, PCR amplification, and sequencing

All specimens were collected in Yunnan Province, Guizhou Province and the Guangxi Zhuang Autonomous Region, China (Suppl. material 1: table S1). Total genomic DNA was extracted from adult leg muscle tissue using the TIANamp Genomic DNA Kit (TIANGEN, Beijing, China) following the manufacturer’s protocols and stored at −20 °C until use.

The standard DNA barcode fragment of mitochondrial cytochrome *c* oxidase subunit I (COI, 658 bp) was amplified with the universal primers LCO1490 and HCO2198 (Folmer et al. 1994). PCR amplification was performed under the following conditions: initial denaturation at 94 °C for 4 min, 35 cycles of denaturation at 94 °C for 30 s, annealing at 48–50 °C for 30 s, an extension at 72 °C for 1 min; followed by a final extension at 72 °C for 5 min. PCR products were verified by 1% agarose gel electrophoresis and sequenced by Sangon Biotech Co. (Shanghai, China).

In addition to direct PCR sequencing, COI sequences were also extracted from mitochondrial genomes generated in this study. For these samples, genomic DNA was extracted from adult prothoracic and leg muscle tissues using the modified CTAB method (Shahjahan et al. 1995). Whole-genome shotgun sequencing was performed at Personal Biotechnology Co., Ltd (Shanghai, China) on the Illumina NovaSeq platform with 150 bp paired-end reads and an average insert size of 350 bp. Raw reads were filtered using Fastp (Chen et al. 2018), yielding approximately 3 Gb of clean data per sample. Mitochondrial genomes were assembled using GetOrganelle v. 1.7.5.0 with k-mer sizes of 21, 45, 65, 85, and 105 and a t-value of 15 (Jin et al. 2020). Gene annotation was conducted using Geneious Prime 2024.0.4 (Kearse et al. 2012). COI sequences were subsequently extracted from the annotated mitogenomes for further analyses.

The NCBI GenBank accession numbers of the sequences used in this study are provided in Suppl. material 1: table S1.

### Phylogenetic analyses

A total of 28 COI sequences were included in the phylogenetic analyses, comprising 27 ingroup sequences representing eight putative species and one outgroup sequence (*Grammographus notabilis cuneatus*).

Sequences were assembled and exported in FASTA format with unique identifiers and aligned using MUSCLE (Edgar 2004) implemented in MEGA v. 7.0 (Kumar et al. 2016). Phylogenetic relationships were reconstructed using neighbor-joining (NJ) and maximum likelihood (ML) methods. The NJ tree was inferred in MEGA v. 7.0 under the Kimura 2-parameter (K2P) model (Kimura 1980) with 1000 bootstrap replicates (Saitou and Nei 1987). The ML tree was reconstructed under the K2P+G+I model, with rate variation among sites modeled using a gamma distribution and a proportion of invariant sites (Yang 1994). Heuristic searches were performed using the Nearest-Neighbor-Interchange (NNI) method (Takahashi and Nei 2000), with initial trees generated automatically using NJ and BioNJ algorithms (Saitou and Nei 1987; Gascuel 1997). Branch support was evaluated with 1000 bootstrap replicates (Felsenstein 1985), and bootstrap values were mapped onto the tree. Interspecific and intraspecific genetic distances were analyzed using COI sequences with the Kimura 2-parameter (K2P) model in MEGA v. 7.0.

## Results

### Phylogenetic analyses and genetic divergence

**Figure 1.**
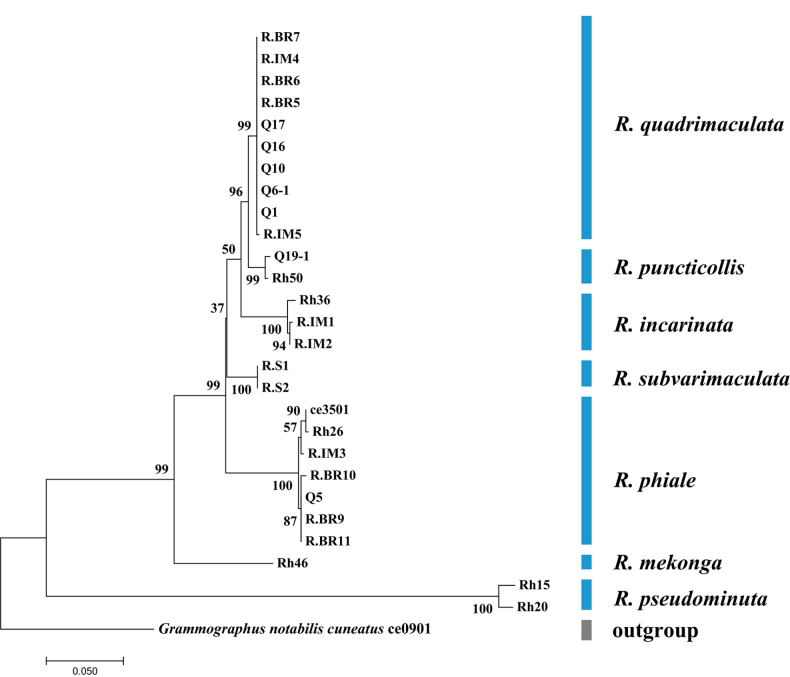
Maximum likelihood (ML) phylogenetic tree based on mitochondrial COI gene sequences of eight putative species of *Rhaphuma*. Bootstrap support values are shown at the nodes.

Phylogenetic analyses based on 27 ingroup COI sequences recovered largely congruent topologies between the neighbor-joining (NJ) and maximum likelihood (ML) methods. The following description focuses specifically on the major topological features of the ML tree (Fig. 1), while the NJ tree is provided in Suppl. material 1: fig. S1.

Both analyses consistently recovered seven well-supported clades (Fig. 1; Suppl. material 1: fig. S1), corresponding to the seven species recognized in this study. Pairwise genetic distances are presented in Suppl. material 1: table S2.

Interspecific genetic distances observed in this study ranged from 1.70% to 22.9%, whereas intraspecific variation was generally low. In *R. quadrimaculata*, intraspecific distances ranged from 0.00% to 0.20%, while in *R. puncticollis* they reached 0.50%. The genetic distances between *R. quadrimaculata* and *R. puncticollis* ranged from 1.70% to 2.00%.

In *R. incarinata*, intraspecific genetic distances ranged from 0.20% to 0.80%. The interspecific genetic distances between *R. puncticollis* and *R. incarinata* ranged from 4.60% to 4.90%. Pairwise genetic distances among *R. quadrimaculata*, *R. puncticollis* and *R. incarinata* ranged from 1.70% to 4.90%.

For *R. phiale*, intraspecific genetic distances ranged from 0.00% to 1.10%, whereas no intraspecific variation was detected in *R. subvarimaculata*. Notably, Rh15 (identified as *R. pseudominuta* Gressitt & Rondon, 1970), and Rh20 (identified as *R. aequalis* Holzschuh, 1991) were recovered as closely related terminals, with a pairwise genetic distance of 1.90%.

### Taxonomy

#### Rhaphuma incarinata

Taxon classificationAnimaliaColeopteraCerambycidae

Pic, 1925

70AF0038-0D4D-584E-80FC-6FB30B68C502

Fig. 2

Rhaphuma incarinata Pic, 1925: 24. Type locality: Laos.

##### Specimens examined.

China • 2♂, Yunnan Province, Jinghong City, Jinuo Mountain Township, Jinuo Mountain, 1100 m, 13–15 May 2018, Qiu Jianyue, Peng Chenli & Xu Hao leg. (SWU) • 1♂, Yunnan Province, Xishuangbanna, Mengla County, Mingshang Tea Planting Agricultural Cooperative, 4 May 2024, Li Zhu & Tian Lichao leg. (SWU).

##### Distribution.

**Figure 2.**
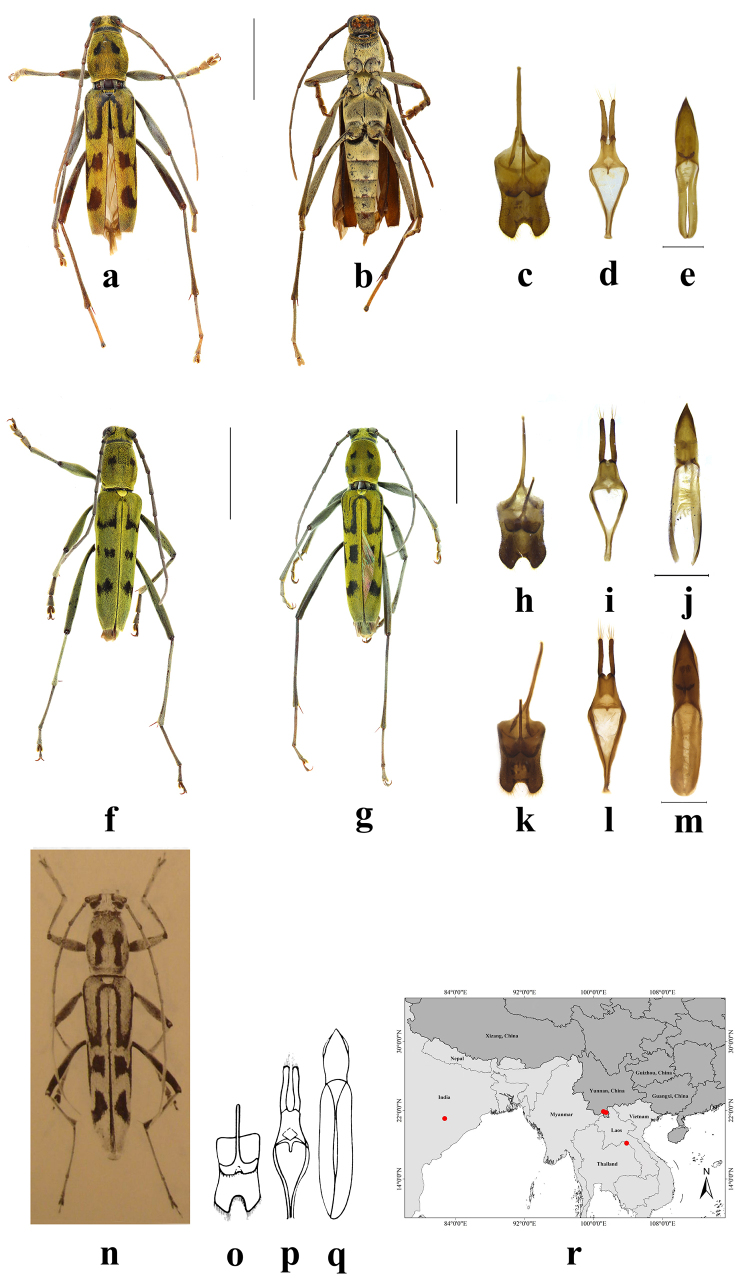
Comparison of *Rhaphuma incarinata* Pic, 1925 with *Rhaphuma improvisa* Holzschuh, 1991. **a**–**m, r**. *R. incarinata*. **a, b, f, g**. Male; **a, f, g**. Dorsal view; **b**. Ventral view; **c**–**e, h**–**m**. Male genitalia, ventral view; **c, h, k**. Tergite VIII with sternites VIII and IX; **d, i, l**. Tegmen; **e, j, m**. Median lobe; **r**. Distribution map; **n**–**q**. *R. improvisa* (from Holzschuh 1991); **n**. Male, holotype; **o**–**q**. Male genitalia; **o**. Tergite VIII with sternites VIII and IX; **p**. Tegmen; **q**. Median lobe. Scale bars: 5 mm (**a**, **b**, **f, g**); 1 mm (**c**–**e**, **h**–**m**); not scaled (**n**–**r**).

China (Yunnan); India, Laos (Fig. 2r). First record from China.

##### Redescription.

Length: 10.8–13.5 mm; width: 2.0–3.0 mm.

Body black to reddish brown, covered with dense yellowish to gray pubescence. Head blackish with yellowish pubescence; antennae reddish brown with pale grayish pubescence. Pronotum with four black spots on the center of the disc, forming a square (Fig. 2a, g), sometimes with only two spots (Fig. 2f). Scutellum blackish, covered with yellowish pubescence. Each elytron with three black markings: 1) U-shaped stripe at basal 1/5 to 1/3; 2) two spots at middle, inner one subsquarish and the outer elongate, often partially fused or with the outer spot reduced in some specimens; and 3) an oblique, slightly curved spot at apical 1/4. Legs reddish brown, femora dark reddish brown, tibiae and tarsi pale reddish brown, covered with grayish pubescence. Ventral surface covered with pale greenish yellow pubescence.

Head short, narrower than pronotum at the widest point. Antennae shorter than body, reaching apical 1/5 of elytra. Antennomere III longer than scape and antennomere IV, antennomere V as long as III, antennomeres VI to XI decreasing slightly in length. Pronotum elongate, 1.25 times as long as the maximum width, rounded at sides; apex distinctly narrower than base; disk slightly convex, coarsely punctate. Scutellum rounded behind. Elytra 3.0 times as long as humeral width, parallel-sided, and slightly narrowed apically; apex subtransversely truncate, disc with fine punctures. Legs slender; femora slightly clavate; tibiae narrow and almost straight; metafemora reaching elytral apex; first metatarsomere 2.0 times as long as remaining tarsomeres combined.

##### Male genitalia.

Tergite VIII longer than wide, with deep U-shaped emargination medially at apical margin, with short or long setae (Fig. 2c, h, k). Tegmen elongate; parameres 1/4 as long as the tegmen, with long setae at each apex (Fig. 2d, i, l). Median lobe short and stout; medianstruts ranges from 3/4 to 5/7 of the length of the median lobe; ventral plate longer and more pointed than dorsal plate (Fig. 2e, j, m).

##### Remarks.

The present identification of *R. incarinata* Pic, 1925 is based on the original description and subsequent interpretation by Holzschuh (1991), as the type material was not examined. The combination of an elongate body, characteristic elytral pattern with a basal arcuate band and median markings, and the presence of four pronotal maculae agrees well with the original concept of the species.

Holzschuh (1991) noted that *R. incarinata* is similar to *R. improvisa*, particularly in the structure of the male genitalia (Holzschuh 1991, fig. 51), but *R. incarinata* can be distinguished by having a simple apex of male sternite VII, shorter hairs on tergite VIII, a shorter basal elytral band, a rectangular punctured area on each side of the pronotum, and the median longitudinal bands of the pronotum usually interrupted medially, forming four distinct spots (Holzschuh 1991). However, our observations indicate that several of these diagnostic characters, especially pronotal bands and the length of setae on tergite VIII, exhibit intraspecific variation and may be of limited taxonomic value.

This raises the possibility that *R. improvisa* and *R. incarinata* may represent a conspecific taxon. However, such a hypothesis cannot be tested at present due to the absence of type material or reliably identified specimens of *R. improvisa*, as well as the lack of molecular data. A taxonomic decision therefore, requires further study.

Some individuals of *R. incarinata* are externally similar to *R. puncticollis* (Fig. 2a, g vs. Fig. 6a, b), but can be reliably distinguished by the male genitalia: tergite VIII with a deep U-shaped emargination (vs. only slightly emarginated in *R. puncticollis*) (Fig. 2c, h, k vs. Fig. 6c, f), and the parameres distinctly more elongate (approximately 7.5 times as long as wide in *R. incarinata* vs. 3.5 times in *R. puncticollis*) (Fig. 2d, i, l vs. Fig. 6d, g).

It can be readily separated from *R. phiale* and *R. quadrimaculata* by the elytral pattern and by distinct differences in the male genitalia (Fig. 2c–e, h–m vs. Figs 4c–e, 7d–f).

#### Rhaphuma mekonga

Taxon classificationAnimaliaColeopteraCerambycidae

Gressitt & Rondon, 1970

107DE0DE-7750-5AA0-A8A1-3D6E927408FE

Fig. 3

Rhaphuma mekonga Gressitt & Rondon, 1970: 237, 251, fig. 38l. Type locality: “Laos, Borikhane Prov., Pakkading”.

##### Specimens examined.

China • 1♂, Guizhou Province, Libo County, Maolan National Nature Reserve, 790 m, 11–17 Jun 2018, Qiu Jianyue & Xu Hao leg. (SWU) • 1♂, Guangxi Zhuang Autonomous Region, Baise City, Leye County, Yachang Township, 1200 m, 29 June 2025, local collector (SWU).

##### Distribution.

**Figure 3.**
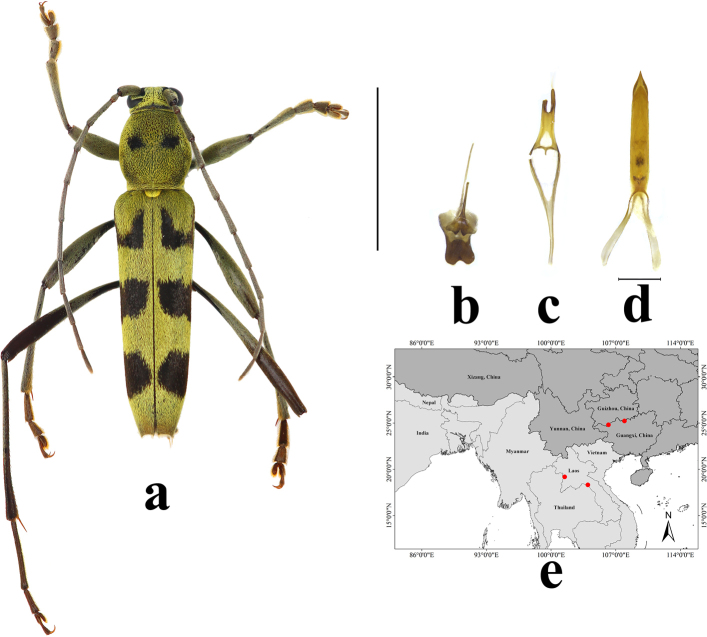
*Rhaphuma mekonga* Gressitt & Rondon, 1970. **a**. Male, dorsal habitus; **b**–**d**. Male genitalia, ventral view; **b**. Tergite VIII with sternites VIII and IX; **c**. Tegmen; **d**. Median lobe; **e**. Distribution map. Scale bars: 5 mm (**a**); 1 mm (**b**–**d**); not scaled (**e**).

China (Guizhou, Guangxi); Laos (Fig. 3e). First record from China.

##### Characteristics.

Length: 11.0–13.0 mm; width: 2.1–2.6 mm. According to Gressitt and Rondon (1970), this species body black, largely clothed with greenish yellow pubescence. Antennae brown with greenish-yellow pubescence. Pronotum black and densely pubescent, with a small blackish spot on each side of center. Elytron with three black bands: 1) one transverse band at basal 1/5; 2) one subtransverse band at middle; and 3) one subrounded spot at apical 1/4. Legs dark brown to black. Antennae reaching 1/8 elytral apex in males and slightly shorter in females. Pronotum as long as wide, slightly wider at base than at apex. Elytra 3.0 times as long as humeral width, elytral apex subobliquely truncated. Legs moderately stout; metafemora reaching beyond elytral apices; first metatarsomere 2.0 times as long as the following two segments combined.

##### Male genitalia.

Tergite VIII longer than wide, narrowly rounded and medially emarginate at apical margin, and with short setae (Fig. 3b). Tegmen long and slender; parameres 1/9 the length of the tegmen, with short setae at each apex (Fig. 3c). Median lobe elongate; median struts 2/5 the length of the entire median lobe; ventral plate longer than dorsal plate (Fig. 3d).

#### Rhaphuma phiale

Taxon classificationAnimaliaColeopteraCerambycidae

Gahan, 1906

2EBD9824-B293-5AED-B4DC-9289B784B1B6

Fig. 4

Rhaphuma phiale Gahan, 1906: 273. Type locality: “Burma: Karen Mts”.

##### Specimens examined.

China • 3♂♂ 1♀, Yunnan Province, Pu’er City, Simao District, Meizi Lake Park, 24 Apr 2014, Huang Guiqiang, Qiu Jianyue, Li Xinran & Feng Xunsi leg. (SWU) • 1♂, Yunnan Province, Pu’er City, Simao District, Meizi Lake Park, 1400 m, 9–11 May 2018, Qiu Jianyue, Peng Chenli & Xu Hao leg. (SWU) • 1♀, Yunnan Province, Pu’er City, Folian Mountain, Mo-Si Highway, 2 May 2021, Tian Lichao leg. (SWU) • 1♂, Yunnan Province, Pu’er City, Mojiang, 30 Apr 2013, Tian Lichao & Huang Guiqiang leg. (SWU) • 1♀, Yunnan Province, Pu’er City, Mojiang, 6 May 2012, Tian Lichao & Huang Guiqiang leg. (SWU) • 1♂, Yunnan Province, Pu’er City, Simao District, 9–11 May 2018, Qiu Jianyue, Peng Chenli & Xu Hao leg. (SWU) • 1♂, Yunnan Province, Pu’er City, Simao District, Caiyang River, 1500 m, 11–13 May 2018, Qiu Jianyue, Peng Chenli & Xu Hao leg. (SWU).

##### Distribution.

**Figure 4.**
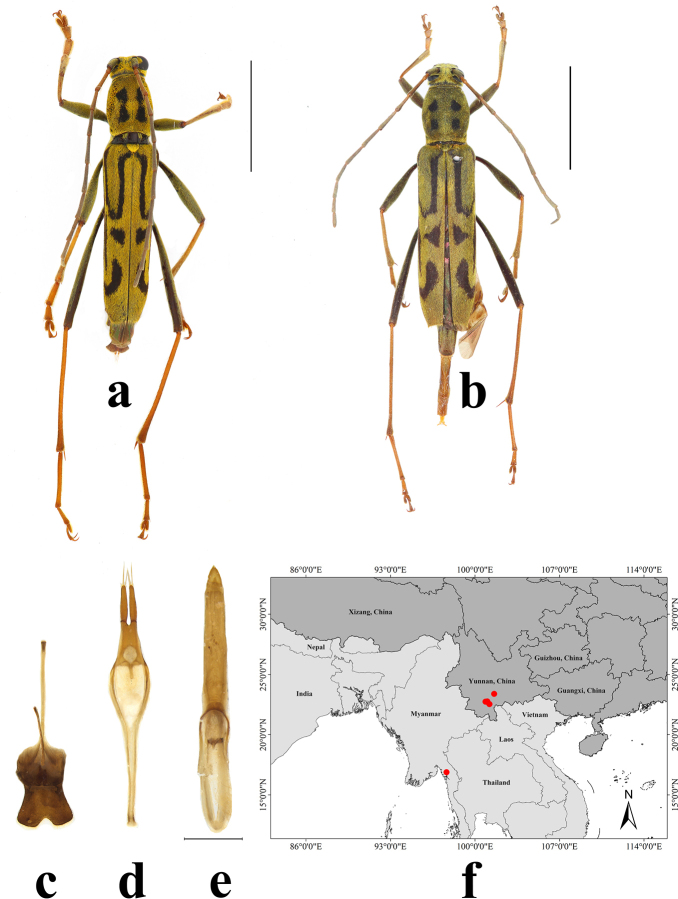
*Rhaphuma phiale* Gahan, 1906. **a, b**. Dorsal habitus; **a**. Male; **b**. Female; **c**–**e**. Male genitalia, ventral view; **c**. Tergite VIII with sternites VIII and IX; **d**. Tegmen; **e**. Median lobe; **f**. Distribution map. Scale bars: 5 mm (**a, b**); 1 mm (**c**–**e**); not scaled (**f**).

China (Yunnan); Myanmar (Fig. 4f).

##### Characteristics.

Length: 12.0–14.0 mm; width: 2.5–3.0 mm. According to Gahan (1906), this species body is black, densely covered with yellow pubescence. Antennae blackish to reddish brown, scape dark brown, somewhat densely covered with greenish-gray pubescence. Pronotum with two short, longitudinal black bands on disc, each sometimes divided into subequal paired spots in our observations. Each elytron with black markings: 1) nearly U-shaped at basal 1/3; 2) a large transverse black spot at middle, sometimes connected with a short lateral stripe; and 3) an oblique, somewhat curved spot at apical 1/4. Femora dark brown, tibiae and tarsi testaceous. Antennae extending to apical 1/4 of elytra in males, to apical 1/3 in females. Pronotum slightly longer than wide, narrowed behind apex. Elytra approximately 3.3 times as long as humeral width, transversely truncate at apex. Metafemora extending beyond elytral apex slightly in males but not exceeding apex in females; first metatarsomere 2.0 times as long as the following two segments combined.

##### Male genitalia.

Tergite VIII longer than wide, truncate and emarginate medially at apical margin (Fig. 4c). Tegmen elongated; parameres nearly 1/6 as long as tegmen, with short setae at each apex (Fig. 4d). Median lobe long and slender, median struts 2/5 as long as entire median lobe; ventral plate longer than dorsal plate; apex of ventral and dorsal plates acutely pointed (Fig. 4e).

##### Remarks.

This species was originally described from Myanmar. Although previously reported from Laos (Gressitt and Rondon 1970) and Hainan, China (Hua et al. 1993), comparative examination of type material accessed via http://bezbycids.com/byciddb/wdetails.asp?id=17184&w=o and a re-examination of the published figures indicate that the record from Laos and Hainan was based on a misidentification. Accordingly, the species is here considered to be reliably recorded in Myanmar and Yunnan Province, China, to date.

#### Rhaphuma pseudominuta

Taxon classificationAnimaliaColeopteraCerambycidae

Gressitt & Rondon, 1970

AAB9DEBB-153A-5871-A89A-FE43BB678605

Fig. 5

Rhaphuma pseudominuta Gressitt & Rondon, 1970: 246, fig. 38f. Type locality: “Laos, NW of Xieng Khouang, Ban Theuong”.
Rhaphuma aequalis Holzschuh, 1991: 49, figs 23, 52. Type locality: “Thailand, Chiang Rai, Wiang Pa Pao”, syn. nov.

##### Specimens examined.

China • 1♂, Yunnan Province, Jinghong City, Jinuo Mountain Township, Jinuo Mountain, 1100 m, 13–15 May 2018, Qiu Jianyue, Peng Chenli & Xu Hao leg. (SWU) • 2♂1♀, Yunnan Province, Xishuangbanna Dai Autonomous Prefecture, Mengla County, Mingshang Tea Planting Agricultural Cooperative, 4 May 2024, Li Zhu & Tian Lichao leg. (SWU).

##### Distribution.

**Figure 5.**
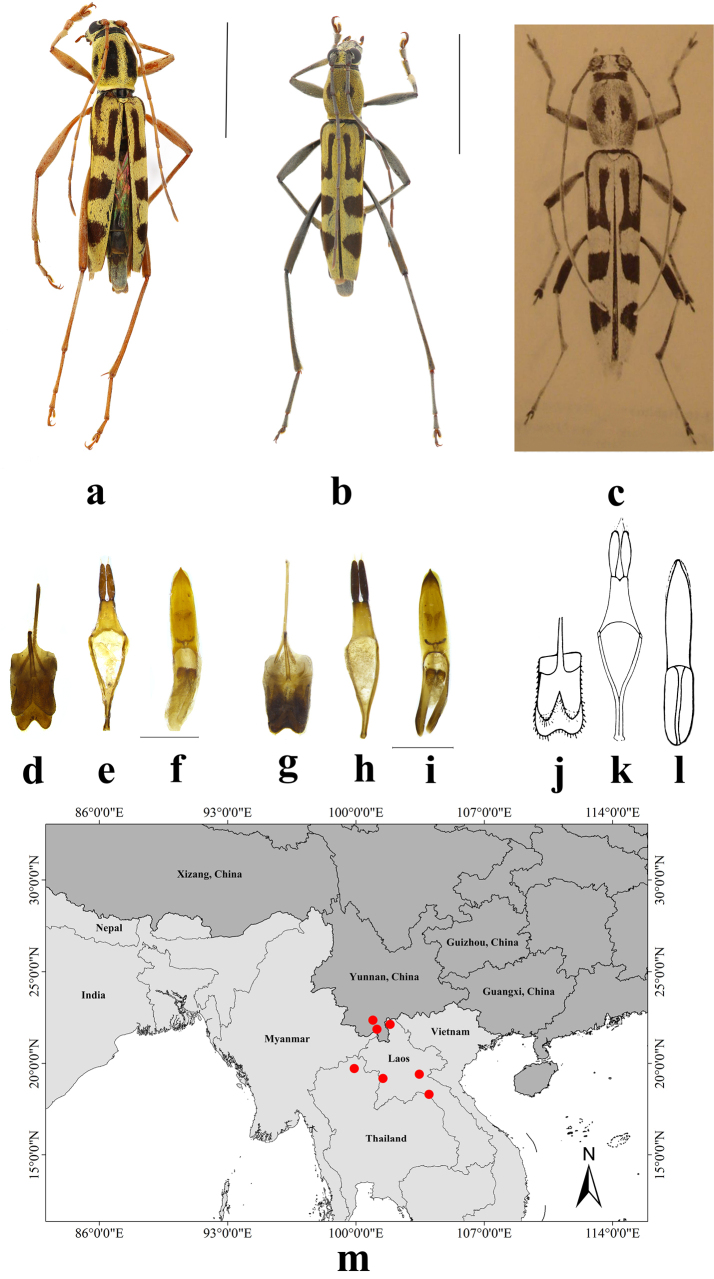
Comparison of *Rhaphuma pseudominuta* Gressitt & Rondon, 1970 with *Rhaphuma aequalis* Holzschuh, 1991. **a, d**–**f**. *R. pseudominuta*; **a**. Male, dorsal habitus; **d**–**f**. Male genitalia, ventral view; **d**. Tergite VIII with sternites VIII and IX; **e**. Tegmen; **f**. Median lobe; **b, c, g–l**. *R. aequalis*; **b, c**. Male, dorsal habitus (**c**. Male holotype); **g**–**l**. Male genitalia, ventral view; **g, j**. Tergite VIII with sternites VIII and IX; **h, k**. Tegmen; **i, l**. Median lobe (**c, j**–**l**. From Holzschuh 1991); **m**. Distribution map. Scale bars: 5 mm (**a, b**); 1 mm **(d**–**i)**; not scaled (**c, j**–**m**).

China (Yunnan); Thailand, Laos (Fig. 5m). First record from China.

##### Redescription.

Length: 8.7–12.9 mm; width: 1.8–2.1 mm. Body pale reddish brown to dark brown, covered with pale yellowish to gray-green pubescence. Head dark brown to reddish brown with ochreous pubescence above. Antennae pale reddish brown or dark brown, covered with fine silvery golden pubescence. Pronotum black with yellowish to grayish pubescence with a broad blackish stripe on disc and a much smaller one on each side (Fig. 5a), or with only one pair of black spots (Fig. 5b). Elytron with following brown markings: 1) a humeral stripe extending to end of basal 1/3, joining a marginal stripe at each end and also continuing transversely on to disc and then forward back almost to scutellum; 2) a transverse band behind middle not reaching suture; 3) a subtriangular spot of apical 1/4; ventral surfaces pitchy with silvery yellow pubescence. Legs reddish brown or black, thinly covered with silvery-buff pubescence.

Head narrowed and finely punctured. Antennae reaching to apical 1/8 of elytra in males, slightly exceeding the middle of elytra in females. Pronotum longer than wide, wider at base than at apex. Scutellum rounded. Elytra 3.6 times as long as humeral width; apex obliquely truncate and outer angle slightly toothed. Legs very slender; metafemora slightly extending beyond the elytral apex; first metatarsomere 2.0 times as long as the following two segments combined.

##### Male genitalia.

Tergite VIII is longer than wide, with a medially emargination on apical margin and bearing short setae (Fig. 5d, g, j). Parameres 1/5 as long as length of tegmen, transversely ridged at each base, with long setae at each apex (Fig. 5e, h, k). Median lobe stout, median struts 1/2 length of entire median lobe; ventral plate longer than dorsal plate, pointed at apex (Fig. 5f, i, l).

##### Remarks.

According to Holzschuh (1991), *R. pseudominuta* and *R. aequalis* are very similar, but the former is distinguished by its reddish-brown legs, antennae, and elytra, as well as by having a much larger longitudinal macula on each side of the median pronotal disc and a small one laterally. In the present study, we examined and sequenced one specimen identified as *R. pseudominuta* (voucher Rh15, Fig. 5a) and one specimen identified as *R. aequalis* (voucher Rh20, Fig. 5b). The identification of Rh20 follows Holzschuh (1991), and is based on the characters mentioned above. However, no reliable differences were found between these two specimens in the male genitalia, and the elytral markings are also very similar. The genetic distance between Rh15 and Rh20 is 1.9% (Suppl. material 1: table S2). Based on the low molecular divergence and the absence of stable diagnostic differences in the male genitalia and elytral markings, we consider the differences in coloration and pronotal markings to represent intraspecific variation. Accordingly, the following synonymy is proposed: *Rhaphuma pseudominuta* Gressitt & Rondon, 1970 = *Rhaphuma aequalis* Holzschuh, 1991, syn. nov.

#### Rhaphuma puncticollis

Taxon classificationAnimaliaColeopteraCerambycidae

Holzschuh, 1992

E61D68E0-EA54-5A8F-B69D-EA80E0763BAE

Fig. 6

Rhaphuma puncticollis Holzschuh, 1992: 34. figs 39, 78. Type locality: “N-Thailand, Chiang Rai, Wiang Pa Pao”.

##### Specimens examined.

China • 1♂, Yunnan Province, Jinghong City, Jinuo Mountain Township, Jinuo Mountain, 1100 m, 13–15 May 2018, Qiu Jianyue, Peng Chenli & Xu Hao leg. (SWU) • 1♂, Yunnan Province, Xishuangbanna, Jinghong City, Dadugang Township, 4 May 2013, Tian Lichao & Huang Guiqiang leg. (SWU).

##### Distribution.

**Figure 6.**
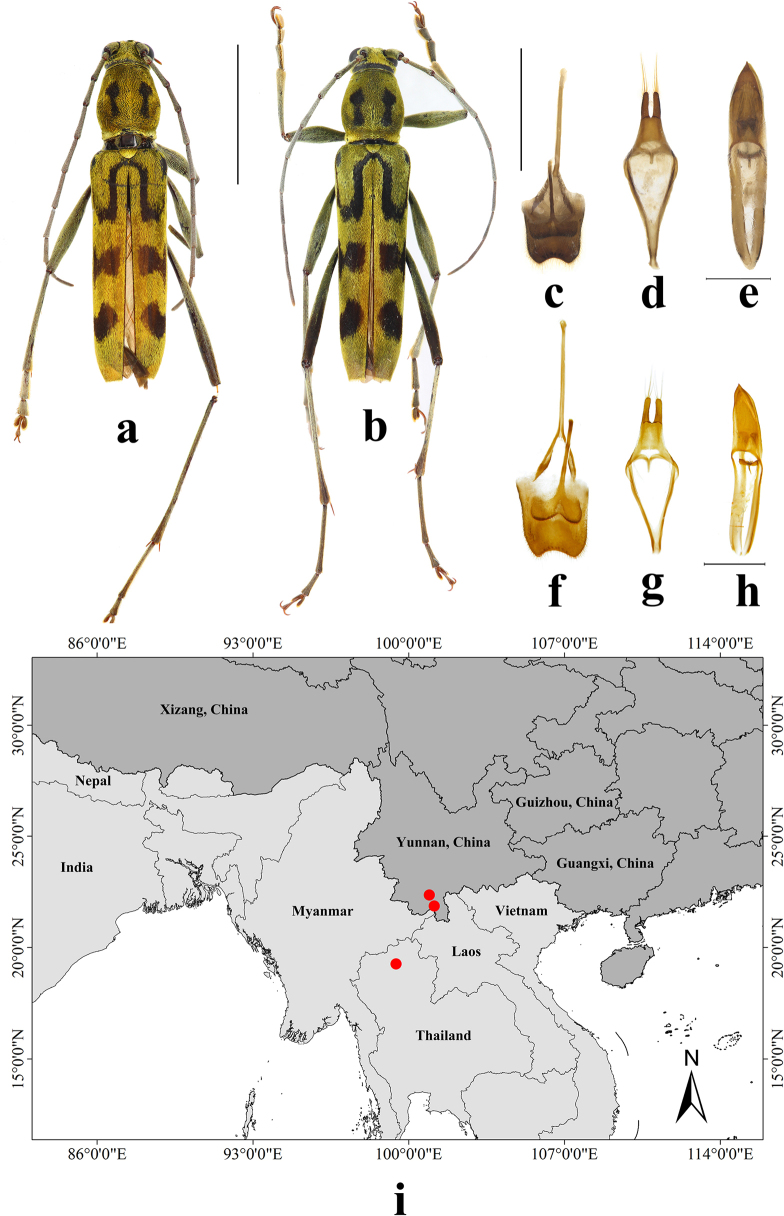
*Rhaphuma puncticollis* Holzschuh, 1992. **a, b**. Male, dorsal habitus; **c**–**h**. Male genitalia, ventral view; **c, f**. Tergite VIII with sternites VIII and IX; **d, g**. Tegmen; **e, h**. Median lobe; **i**. Distribution map. Scale bars: 5 mm (**a, b**); 1 mm (**c**–**h**); not scaled (**i**).

China (Yunnan); Thailand (Fig. 6i).

##### Characteristics.

Length: 8.0–14.5 mm; width: 1.8–3.0 mm. According to Holzschuh (1992), this species has a body black, densely covered with yellow pubescence. Antennae dark brown, covered with grayish pubescence. Pronotum with four black spots, the spots sometimes connected into two short longitudinal bands. Elytra dark reddish brown, each elytron with black markings: 1) a U-shaped stripe at basal 1/4; 2) a median transverse band broadly interrupted at the middle and near the lateral margin or a transverse band narrowed in the middle, but not reaching lateral margin, and 3) an oblique spot at apical 1/3. Legs reddish brown. Antennae reaching apical 1/4 of elytra in males. Pronotum slightly longer than wide, wider at base than at apex. Elytra 3.0 times as long as humeral width; apex truncate. Metafemora slender, extending elytral apex; first metatarsomere 2.0 times as long as the following two segments combined.

##### Male genitalia.

Tergite VIII slightly longer than wide, narrowly rounded and medially emarginate at apical margin, with short setae (Fig. 6c, f). Parameres 1/7 as long as length of tegmen, transversely ridged at each base, with long setae at each apex (Fig. 6d, g). Median lobe stout, median struts 3/5 length of median lobe; ventral plate longer than dorsal plate, pointed at apex (Fig. 6e, h).

##### Remarks.

The genetic distance between *R. puncticollis* and *R. quadrimaculata* is relatively low, ranging from 1.70% to 2.00%. However, a low COI divergence alone is not treated here as evidence of conspecificity. Multiple specimens were sequenced, and more than one male specimen was dissected. The two species can be distinguished by clear differences in the male genitalia, especially in tergite VIII and the tegmen. Therefore, despite the low COI divergence, we prefer to retain *R. puncticollis* and *R. quadrimaculata*.

#### Rhaphuma quadrimaculata

Taxon classificationAnimaliaColeopteraCerambycidae

Pic, 1923

1F654D1A-30C6-5252-A1F7-8C6031B65275

Fig. 7

Rhaphuma *4-maculata* Pic, 1923: 10. Type locality: “Laos”.
Rhaphuma quadrimaculata : Gressitt & Rondon, 1970: 236, 245, Fig. 38d.

##### Specimens examined.

China • 6♂♂ 5♀♀, Yunnan Province, Jinghong City, Jinuo Mountain Township, Jinuo Mountain, 1000 m, 13–15 May 2018, Qiu Jianyue, Peng Chenli & Xu Hao leg. (SWU) • 1♀, Yunnan Province, Pu’er City, Simao District, Caiyang River, 1500 m, 11–13 May 2018, Qiu Jianyue, Peng Chenli & Xu Hao leg. (SWU) • 2♂♂ 2♀♀, Yunnan Province, Pu’er City, Yunpan Mountain, 3 May 2013, Tian Lichao & Huang Guiqiang leg. (SWU) • 1♀, Yunnan Province, Xishuangbanna, Jinghong City, Dadugang Township, Jiejilin Village, 28 Apr 2014, Huang Guiqiang, Qiu Jianyue, Li Xinran & Feng Xunsi leg. (SWU).

##### Distribution.

**Figure 7.**
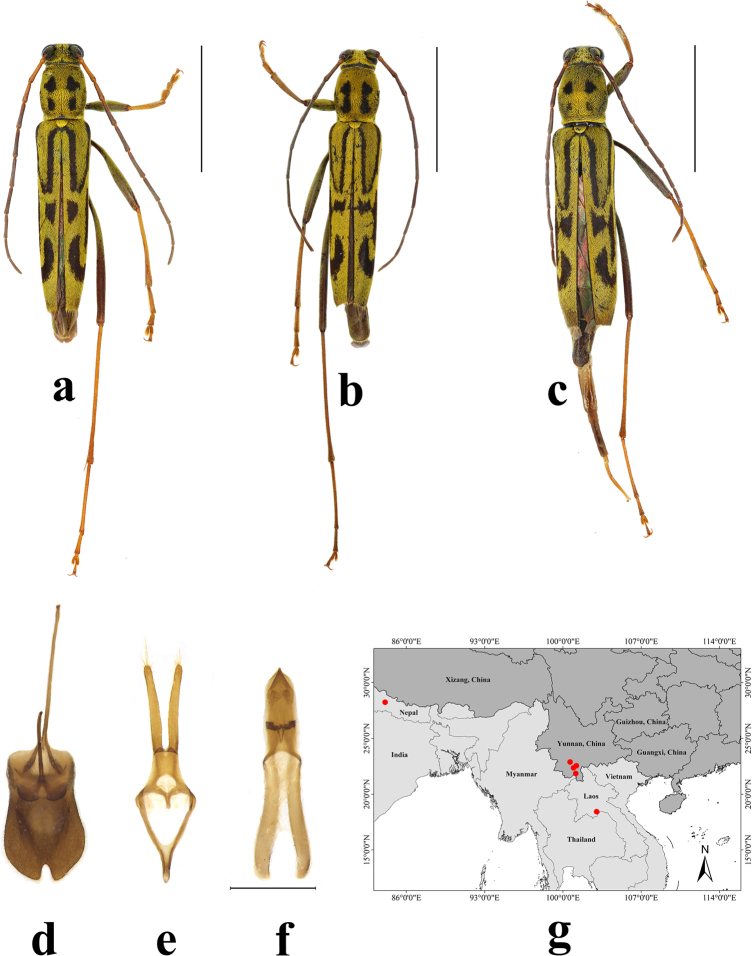
*Rhaphuma quadrimaculata* Pic, 1923. **a**–**c**. Dorsal habitus; **a, b**. Male; **c**. Female; **d**–**f**. Male genitalia, ventral view; **d**. Tergite VIII with sternites VIII and IX; **e**. Tegmen; **f**. Median lobe; **g**. Distribution map. Scale bars: 5 mm (**a**–**c**); 1 mm (**d**–**f**); not scaled (**g**).

China (Yunnan); Nepal, Laos (Fig. 7g).

##### Characteristics.

Length: 11.0–15.0 mm; width: 2.1–2.9 mm. Body black, covered with yellowish pubescence. Antennae testaceous to reddish brown, scape dark brown, with greenish-yellow pubescence. Pronotum with four black spots on center of disc, in some specimens, these spots connected with each other forming two short longitudinal bands. Elytra markings similar to those of *R. phiale*. Legs with dark brown femora and yellowish-brown tibiae and tarsi. Antennae extending to apical 1/4 of elytra in males, to apical 1/3 in females. Pronotum nearly as long as wide, slightly wider at base than at apex. Elytra more than three times as long as humeral width, transversely truncate at apex. Metafemora reaching elytral apex; first metatarsomere 2.0 times as long as remaining tarsomeres combined.

##### Male genitalia.

Tergite VIII longer than wide, rounded and emarginate medially at apical margin (Fig. 7d). Parameres 2/5 as long as length of tegmen, with short setae at each apex (Fig. 7e). Median lobe short; median struts 3/5 length of entire median lobe; ventral plate and dorsal plate almost same in length, ventral plate pointed at apex (Fig. 7f).

##### Remarks.

This species exhibits considerable similarity in general morphology to *R. phiale* Gahan, although it possesses distinctly different male genitalia. The key to *Rhaphuma* provided by Gressitt and Rondon (1970) diagnoses this species by having a “Pronotum with 4 distinct black spots forming a square, without lateral spot”. However, our examination reveals variation in the pronotal maculation patterns across both species (Figs 4a, b, 7a–c).

#### Rhaphuma subvarimaculata

Taxon classificationAnimaliaColeopteraCerambycidae

Gressitt & Rondon, 1970

51CF3801-2FF2-5CB6-A311-E876E7E681C5

Fig. 8

Rhaphuma subvarimaculata Gressitt & Rondon, 1970: 236, 245, fig. 38e. Type locality: “Laos, Vientiane Prov., Ban Van Heua”.

##### Specimens examined.

China • 1♂ 1♀, Yunnan Province, Jinghong City, Jinuo Mountain Township, Jinuo Mountain, 1000 m, 13–15 May 2018, Qiu Jianyue, Peng Chenli & Xu Hao leg. (SWU) • 3♂♂, Yunnan Province, Xishuangbanna, Menglun County, 26 Apr 2016, Qiu Jianyue leg. (SWU).

##### Distribution.

**Figure 8.**
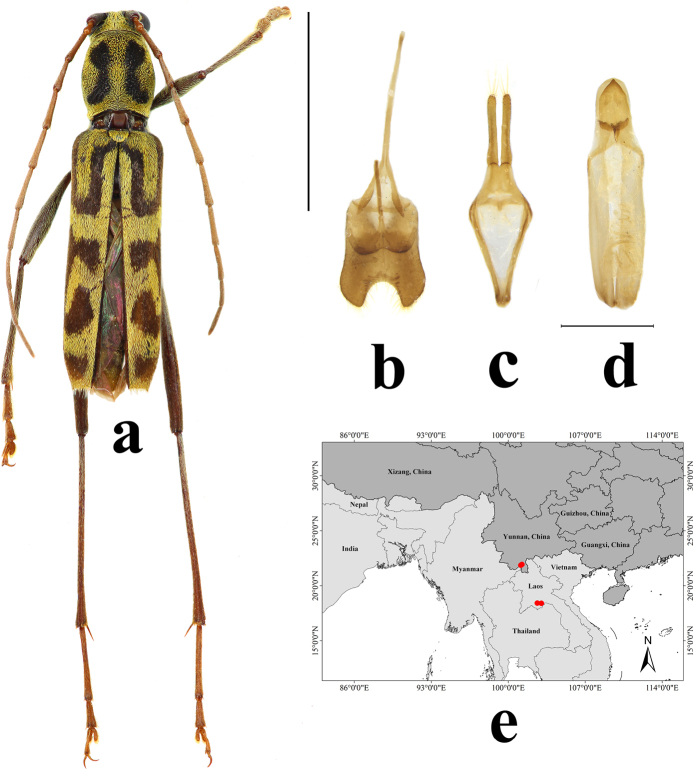
*Rhaphuma subvarimaculata* Gressitt & Rondon, 1970. **a**. Male, dorsal habitus; **b**–**d**. Male genitalia, ventral view; **b**. Tergite VIII with sternites VIII and IX; **c**. Tegmen; **d**. Median lobe; **e**. Distribution map. Scale bars: 5 mm (**a**); 1 mm (**b**–**d**); not scaled (**e**).

China (Yunnan); Laos (Fig. 8e).

##### Characteristics.

Length: 9.5–13.4 mm; width: 2.0–2.5 mm. According to Gressitt and Rondon (1970), this species body black to reddish, with yellowish pubescence. Antennae reddish brown, black on most of scape, with thin golden-gray pubescence. Pronotum black, with yellowish pubescence, with a pair of large )(-shaped marks on disc and a small elliptical spot on side. Each elytron with dark brown markings: 1) a U-shaped stripe at basal 1/3; 2) a transverse band consisting of a triangular spot at basal half, connecting with a longitudinal stripe laterally; 3) an oblique subtriangular spot at apical 1/3; and 4) a subtriangular spot before apex. Legs reddish, darker on much of femora. Antennae reaching to elytral apex in males, reaching apical 1/3 in females. Pronotum slightly longer than wide, narrower at apex than at base. Elytra 2.5 times as long as humeral width, subtransversely truncate at apex. Metafemora reaching beyond elytral apex; first metatarsomere 2.0 times as long as the following two segments combined.

##### Male genitalia.

Tergite VIII longer than wide, rounded and with a distinct U-shaped emargination medially at apical margin (Fig. 8b). Parameres 1/3 as long as tegmen, with long setae at each apex (Fig. 8c). Median struts 3/4 length of entire median lobe; ventral plate as long as dorsal plate, pointed at apex (Fig. 8d).

## Supplementary Material

XML Treatment for Rhaphuma incarinata

XML Treatment for Rhaphuma mekonga

XML Treatment for Rhaphuma phiale

XML Treatment for Rhaphuma pseudominuta

XML Treatment for Rhaphuma puncticollis

XML Treatment for Rhaphuma quadrimaculata

XML Treatment for Rhaphuma subvarimaculata

## Additional information

### Artificial Intelligence (AI) use

Regarding the use of AI in the preparation of this manuscript, the authors declare the following:

The language in certain passages of this article was polished using an AI-powered tool ([e.g., DeepSeek]).

### Author ORCIDs

L. Chen https://orcid.org/0009-0004-5191-8935

Y. Qi https://orcid.org/0009-0008-6015-9335

X. Wang https://orcid.org/0009-0006-7225-4677

C. Jia https://orcid.org/0009-0007-9269-1126

Z. Li https://orcid.org/0000-0002-7322-5863

### Data availability

All of the data that support the findings of this study are available in the main text or Supplementary Information.

## Supplementary materials

Supplementary material 1 Supplementary figure and tables Data type docx Explanation note **fig. S1**. Neighbor-Joining (NJ) phylogenetic tree based on mitochondrial COI gene sequences of eight putative species of *Rhaphuma*. **table S1**. Information about samples used in this study and their NCBI GenBank accession numbers. **table S2**. Interspecific genetic distance based on *COI* sequence (K2P model). https://binary.pensoft.net/file/1694542This dataset is made available under the Open Database License (http://opendatacommons.org/licenses/odbl/1.0/). The Open Database License (ODbL) is a license agreement intended to allow users to freely share, modify, and use this Dataset while maintaining this same freedom for others, provided that the original source and author(s) are credited. Lu Chen, Ye Qi, Xiaoyi Wang, Congrong Jia, Zhu Li
