# Supplementary material for: Taxonomic notes on the genus Rhaphuma Pascoe, 1858 (Coleoptera, Cerambycidae) in China, with focus on the species complex of Rhaphuma incarinata Pic, 1925
Source: Zookeys. 2026 Jun 24;1283:47–65. doi: 10.3897/zookeys.1283.170982 (PMC13324406; doi:10.3897/zookeys.1283.170982)
Supplement: Supplementary material 1 — Supplementary figure and tables [file zookeys-1283-047_article-170982__-s001.docx]

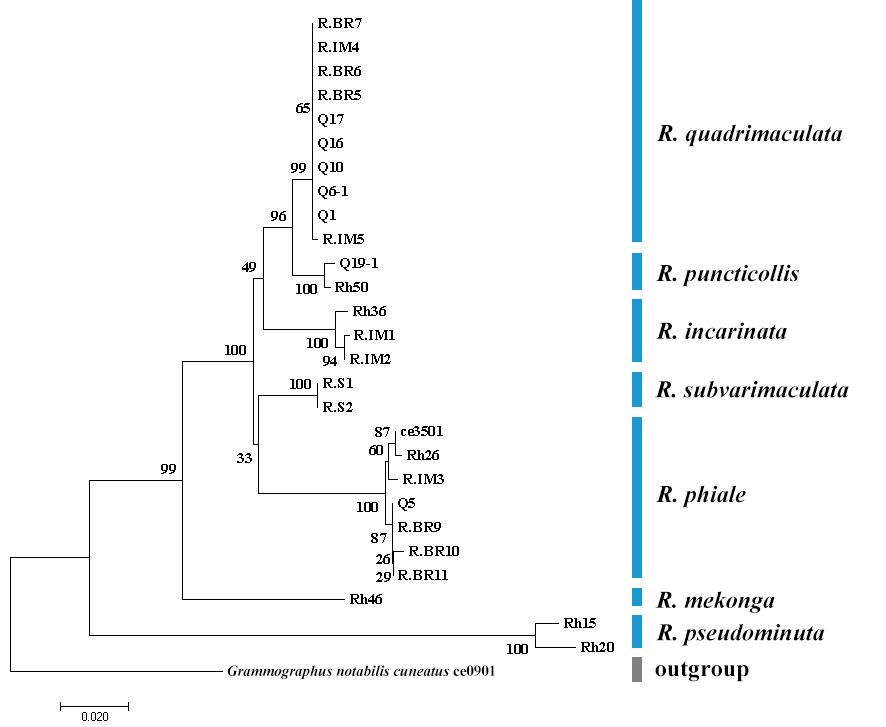


**Figure S1.** Neighbor-Joining (NJ) phylogenetic tree based on mitochondrial *COI* gene sequences of eight putative species of *Rhaphuma*. Bootstrap support values are shown at the nodes.

**Table S1**. Information about samples used in this study and their NCBI GenBank accession numbers.

| **Species** | **voucher specimen number** | **Sample Locality** | **GenBank**  **Accession** | **Reference** |
| --- | --- | --- | --- | --- |
| *R. quadrimaculata* | R.BR5 | Yunnan | PZ325635 | This study |
| *R. quadrimaculata* | R.BR6 | Yunnan | PZ325636 | This study |
| *R. quadrimaculata* | R.BR7 | Yunnan | PZ325637 | This study |
| *R. quadrimaculata* | Q1 | Yunnan | PZ325628 | This study |
| *R. quadrimaculata* | Q6-1 | Yunnan | PZ325630 | This study |
| *R. quadrimaculata* | Q10 | Yunnan | PZ325631 | This study |
| *R. quadrimaculata* | Q16 | Yunnan | PZ325632 | This study |
| *R. quadrimaculata* | Q17 | Yunnan | PZ325633 | This study |
| *R. quadrimaculata* | R.IM4 | Yunnan | PZ325644 | This study |
| *R. quadrimaculata* | R.IM5 | Yunnan | PZ325645 | This study |
| *R. puncticollis* | Q19-1 | Yunnan | PZ325634 | This study |
| *R.* *puncticollis* | Rh50 | Yunnan | PZ325627 | This study |
| *R.* *incarinata* | Rh36 | Yunnan | PZ325625 | This study |
| *R.* *incarinata* | R.IM1 | Yunnan | PZ325641 | This study |
| *R.* *incarinata* | R.IM2 | Yunnan | PZ325642 | This study |
| *R. subvarimaculata* | R.S1 | Yunnan | PZ325646 | This study |
| *R. subvarimaculata* | R.S2 | Yunnan | PZ325647 | This study |
| *R. phiale* | ce3501 | Yunnan | PZ325620 | This study |
| *R. phiale* | Rh26 | Yunnan | PZ325624 | This study |
| *R. phiale* | R.IM3 | Yunnan | PZ325643 | This study |
| *R. phiale* | Q5 | Yunnan | PZ325629 | This study |
| *R. phiale* | R.BR9 | Yunnan | PZ325638 | This study |
| *R. phiale* | R.BR10 | Yunnan | PZ325639 | This study |
| *R. phiale* | R.BR11 | Yunnan | PZ325640 | This study |
| *R. mekonga* | Rh46 | Guizhou | PZ325626 | This study |
| *R. pseudominuta* | Rh15 | Yunnan | PZ325622 | This study |
| *R. pseudominuta* | Rh20 | Yunnan | PZ325623 | This study |
| *Grammographus notabilis cuneatus* | ce0901 | Yunnan | PZ325621 | This study |

**Table S2.** Interspecific genetic distance based on *COI* sequence (K2P model)

| Species | ce3501 | *Grammographusnotabilis cuneatus* ce0901 | Q1 | Q5 | Q6-1 | Q10 | Q16 | Q17 | Q19-1 | R.BR5 | R.BR6 | R.BR7 | R.BR9 |
| --- | --- | --- | --- | --- | --- | --- | --- | --- | --- | --- | --- | --- | --- |
| ce3501 |  |  |  |  |  |  |  |  |  |  |  |  |  |
| *Grammographusnotabilis cuneatus* ce0901 | 0.174 |  |  |  |  |  |  |  |  |  |  |  |  |
| Q1 | 0.059 | 0.151 |  |  |  |  |  |  |  |  |  |  |  |
| Q5 | 0.006 | 0.168 | 0.059 |  |  |  |  |  |  |  |  |  |  |
| Q6-1 | 0.059 | 0.151 | 0.000 | 0.059 |  |  |  |  |  |  |  |  |  |
| Q10 | 0.059 | 0.151 | 0.000 | 0.059 | 0.000 |  |  |  |  |  |  |  |  |
| Q16 | 0.059 | 0.151 | 0.000 | 0.059 | 0.000 | 0.000 |  |  |  |  |  |  |  |
| Q17 | 0.059 | 0.151 | 0.000 | 0.059 | 0.000 | 0.000 | 0.000 |  |  |  |  |  |  |
| Q19-1 | 0.066 | 0.150 | 0.019 | 0.062 | 0.019 | 0.019 | 0.019 | 0.019 |  |  |  |  |  |
| R.BR5 | 0.059 | 0.151 | 0.000 | 0.059 | 0.000 | 0.000 | 0.000 | 0.000 | 0.019 |  |  |  |  |
| R.BR6 | 0.059 | 0.151 | 0.000 | 0.059 | 0.000 | 0.000 | 0.000 | 0.000 | 0.019 | 0.000 |  |  |  |
| R.BR7 | 0.059 | 0.151 | 0.000 | 0.059 | 0.000 | 0.000 | 0.000 | 0.000 | 0.019 | 0.000 | 0.000 |  |  |
| R.BR9 | 0.006 | 0.168 | 0.059 | 0.000 | 0.059 | 0.059 | 0.059 | 0.059 | 0.062 | 0.059 | 0.059 | 0.059 |  |
| R.BR10 | 0.009 | 0.172 | 0.062 | 0.003 | 0.062 | 0.062 | 0.062 | 0.062 | 0.066 | 0.062 | 0.062 | 0.062 | 0.003 |
| R.BR11 | 0.006 | 0.168 | 0.059 | 0.000 | 0.059 | 0.059 | 0.059 | 0.059 | 0.062 | 0.059 | 0.059 | 0.059 | 0.000 |
| R.IM1 | 0.064 | 0.160 | 0.039 | 0.064 | 0.039 | 0.039 | 0.039 | 0.039 | 0.049 | 0.039 | 0.039 | 0.039 | 0.064 |
| R.IM2 | 0.064 | 0.160 | 0.038 | 0.064 | 0.038 | 0.038 | 0.038 | 0.038 | 0.048 | 0.038 | 0.038 | 0.038 | 0.064 |
| R.IM3 | 0.005 | 0.174 | 0.059 | 0.005 | 0.059 | 0.059 | 0.059 | 0.059 | 0.066 | 0.059 | 0.059 | 0.059 | 0.005 |
| R.IM4 | 0.059 | 0.151 | 0.000 | 0.059 | 0.000 | 0.000 | 0.000 | 0.000 | 0.019 | 0.000 | 0.000 | 0.000 | 0.059 |
| R.IM5 | 0.061 | 0.151 | 0.002 | 0.061 | 0.002 | 0.002 | 0.002 | 0.002 | 0.020 | 0.002 | 0.002 | 0.002 | 0.061 |
| R.S1 | 0.059 | 0.158 | 0.036 | 0.056 | 0.036 | 0.036 | 0.036 | 0.036 | 0.039 | 0.036 | 0.036 | 0.036 | 0.056 |
| R.S2 | 0.059 | 0.158 | 0.036 | 0.056 | 0.036 | 0.036 | 0.036 | 0.036 | 0.039 | 0.036 | 0.036 | 0.036 | 0.056 |
| Rh15 | 0.220 | 0.223 | 0.202 | 0.225 | 0.202 | 0.202 | 0.202 | 0.202 | 0.208 | 0.202 | 0.202 | 0.202 | 0.225 |
| Rh20 | 0.218 | 0.227 | 0.213 | 0.223 | 0.213 | 0.213 | 0.213 | 0.213 | 0.206 | 0.213 | 0.213 | 0.213 | 0.223 |
| Rh26 | 0.002 | 0.176 | 0.061 | 0.008 | 0.061 | 0.061 | 0.061 | 0.061 | 0.068 | 0.061 | 0.061 | 0.061 | 0.008 |
| Rh36 | 0.066 | 0.162 | 0.039 | 0.066 | 0.039 | 0.039 | 0.039 | 0.039 | 0.049 | 0.039 | 0.039 | 0.039 | 0.066 |
| Rh46 | 0.110 | 0.155 | 0.087 | 0.109 | 0.087 | 0.087 | 0.087 | 0.087 | 0.087 | 0.087 | 0.087 | 0.087 | 0.109 |
| Rh50 | 0.068 | 0.152 | 0.017 | 0.064 | 0.017 | 0.017 | 0.017 | 0.017 | 0.005 | 0.017 | 0.017 | 0.017 | 0.064 |

**Table S2.** Interspecific genetic distance based on *COI* sequence (K2P model)

| Species | R.BR10 | R.BR11 | R.IM1 | R.IM2 | R.IM3 | R.IM4 | R.IM5 | R.S1 | R.S2 | Rh15 | Rh20 | Rh26 | Rh36 | Rh46 | Rh50 |
| --- | --- | --- | --- | --- | --- | --- | --- | --- | --- | --- | --- | --- | --- | --- | --- |
| R.BR10 |  |  |  |  |  |  |  |  |  |  |  |  |  |  |  |
| R.BR11 | 0.003 |  |  |  |  |  |  |  |  |  |  |  |  |  |  |
| R.IM1 | 0.067 | 0.064 |  |  |  |  |  |  |  |  |  |  |  |  |  |
| R.IM2 | 0.068 | 0.064 | 0.002 |  |  |  |  |  |  |  |  |  |  |  |  |
| R.IM3 | 0.008 | 0.005 | 0.068 | 0.068 |  |  |  |  |  |  |  |  |  |  |  |
| R.IM4 | 0.062 | 0.059 | 0.039 | 0.038 | 0.059 |  |  |  |  |  |  |  |  |  |  |
| R.IM5 | 0.064 | 0.061 | 0.041 | 0.039 | 0.061 | 0.002 |  |  |  |  |  |  |  |  |  |
| R.S1 | 0.059 | 0.056 | 0.054 | 0.053 | 0.059 | 0.036 | 0.034 |  |  |  |  |  |  |  |  |
| R.S2 | 0.059 | 0.056 | 0.054 | 0.053 | 0.059 | 0.036 | 0.034 | 0.000 |  |  |  |  |  |  |  |
| Rh15 | 0.229 | 0.225 | 0.210 | 0.208 | 0.225 | 0.202 | 0.202 | 0.208 | 0.208 |  |  |  |  |  |  |
| Rh20 | 0.227 | 0.223 | 0.221 | 0.219 | 0.223 | 0.213 | 0.213 | 0.206 | 0.206 | 0.019 |  |  |  |  |  |
| Rh26 | 0.011 | 0.008 | 0.066 | 0.066 | 0.006 | 0.061 | 0.062 | 0.061 | 0.061 | 0.223 | 0.221 |  |  |  |  |
| Rh36 | 0.069 | 0.066 | 0.008 | 0.006 | 0.070 | 0.039 | 0.041 | 0.051 | 0.051 | 0.206 | 0.216 | 0.068 |  |  |  |
| Rh46 | 0.113 | 0.109 | 0.095 | 0.095 | 0.111 | 0.087 | 0.088 | 0.088 | 0.088 | 0.216 | 0.223 | 0.111 | 0.094 |  |  |
| Rh50 | 0.067 | 0.064 | 0.047 | 0.046 | 0.068 | 0.017 | 0.019 | 0.041 | 0.041 | 0.208 | 0.206 | 0.069 | 0.047 | 0.085 |  |
